# A prospective study of hand grip strength and cardiovascular outcomes in a cardiovascular intensive care unit

**DOI:** 10.3389/fcvm.2025.1677500

**Published:** 2026-01-05

**Authors:** Binaya Basyal, Harish Jarrett, Neha Gupta, Phillip Nelson, Evan Czulada, Alexandra A. Taylor, Caroline E. Adams, Allen J. Taylor

**Affiliations:** 1 Department of Cardiology, MedStar Heart and Vascular Institute and Georgetown University School of Medicine, Washington, DC, United States; 2 Lankenau Heart Institute, Wynnewood, PA, United States

**Keywords:** hand grip strength, cardiovascular intensive care, frailty, outcome study, length of stay

## Abstract

**Background:**

Handgrip strength (HGS) is a marker of frailty that is associated with major adverse cardiovascular outcomes. The relationship between HGS and outcomes in a cardiovascular intensive care unit (CVICU) setting has not been previously studied.

**Methods:**

We measured handgrip strength upon admission to the CVICU among 330 consecutive adult patients. Subsequent clinical outcomes of interest included readmission to the CVICU, CVICU and hospital length of stay (LOS), 30-day hospital readmission, and in-hospital mortality. Mean values were compared using the student t-test and Pearson's r was used to test bivariate correlation.

**Results:**

330 patients underwent HGS assessment. HGS was significantly inversely correlated with hospital LOS (r = −0.165, *P* = 0.003) and mean LOS was 3 days longer among the lowest quartile (HGS <18 kg; *P* = 0.049). HGS was not associated with either CVICU or 30-day readmission and mortality. Among non-procedural admissions to the CVICU, linear regression identified HGS, age, and albumin as significant predictors of hospital LOS (r = 0.38; *P* < 0.001). Following an elective procedure, the Oxford Acute Severity of Illness Score (OASIS) score (r = 0.426, *P* < 0.001) and albumin (r = −0.380, *p* < 0.001) were better predictors of LOS than HGS.

**Conclusions:**

Hand grip strength provides a simple point of care assessment in the CVICU for determination of patient frailty. Lower values are independently associated with hospital length of stay among non-procedural patients.

## Introduction

The modern cardiovascular intensive care unit (CVICU) has evolved from providing rapid resuscitation to a place where comprehensive critical care is provided (1). Advancing age and severe multisystem comorbid illness commonly co-exist alongside cardiovascular disease serving to increase the complexity of patients treated in the CVICU (2). In addition, advances in treating valvular heart disease, cardiac arrhythmias and the utilization of increasingly complex mechanical circulatory support systems have fundamentally changed the case mix in the contemporary CVICU (3).

Intensive care unit (ICU) hospitalization can have a negative impact on an individual's quality of life up to 5 years after hospital discharge (4). Functional and psychological limitations appear to persist with associated increases in cost and utilization of health care services (5). Intensive care unit acquired weakness (ICUAW), defined as a clinically detectable weakness that is attributable to the critical illness itself (6), appears to play a key role in this long-term disability syndrome. ICUAW appears to be remarkably common with an incidence of 40% (7) and is associated with higher morbidity, mortality and longer length of stay (8). Identification of patients at risk for ICUAW and its accompanying adverse outcomes is critical to deploy the necessary therapeutic strategies in a timely fashion.

Among various indices of frailty, bedside assessment of hand-grip strength (HGS) is an accurate and reliable (4) correlate of global muscle strength that predicts length of stay as well as mortality in critically ill medically treated ICU patients (9). Numerous epidemiological studies have shown that lower hand grip strength is associated with increased risk of functional limitations and disability, increased long term mortality, higher activities of daily living dependence, cause specific and total mortality, cardiovascular mortality and respiratory mortality (10). Low HGS is a marker of the frailty phenotype (11) which is predictive of risk of falls, immobility, hospitalizations, and death in a community-dwelling cohort aged >65 years. HGS has been studied as a marker of old age disability (12) and nutritional status. Low HGS has also been associated with an increased risk of all-cause and cardiovascular mortality, myocardial infarction and stroke (13). No study to date has examined HGS in the CVICU patient population, and the relationship between baseline HGS and outcomes following admission to the CVICU is not known. We aimed to describe normal distribution of HGS in a cardiovascular intensive care unit, compare HGS to established validated clinical frailty scales and describe the relationship between HGS and ICU length of stay (LOS), mortality and readmission.

## Methods

### Study design

This prospective cohort study was conducted in consecutive patients over a 6 month period from December 2018 to May 2019 who were admitted at our institution's CVICU. The study was approved by the institutional review board and all patients provided written informed consent to participate in the study.

### Inclusion and exclusion criteria

All patients over the age of 18 years admitted to CVICU who were able to participate in HGS assessment were included in the study. We excluded patients who had history of dementia, experienced delirium in the past 24 h, were not able to speak and/or understand English, not oriented to time, place and person and who declined consent to participate. Subjects who were sedated and unable to cooperate with HGS testing (mechanically ventilated, paralyzed, or delirious) underwent HGS assessment on the first day of their CVICU stay in which they could cooperate. Eligible subjects included patients admitted for acute medical conditions and those admitted following elective cardiovascular (nonsurgical) procedures.

### Assessment of hand grip strength and clinical variables

**Figure 1:**
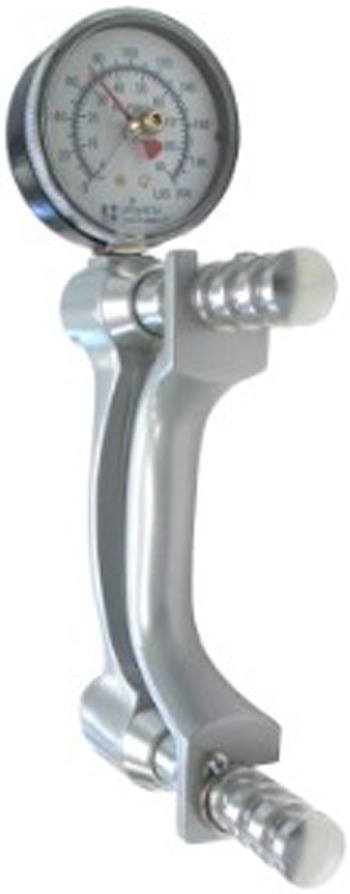
Lafayette hand dynamometer. (Lafayette Instruments, Lafayette IN).

Patients admitted to the CVICU underwent HGS testing using a Lafayette hand dynamometer (Figure 1; Lafayette, IN) on the first day of their CVICU stay using a standardized protocol. Subjects were asked to perform a maximal isometric contraction with each hand three consecutive times with each contraction followed by a 5 s rest period. Both peak grip strength and averages measured in pounds for each hand were taken. For each patient we collected baseline demographic, clinical and laboratory data, calculated Charlson comorbidity index and assessed severity of illness using Oxford Acute Severity of Illness Score (OASIS).

### Outcomes

The primary outcome of interest was overall hospital length of stay. Secondary endpoints included CVICU LOS, mortality, and readmission to either the CVICU or hospital at 30 days.

### Statistical analysis

All analyses were conducted using SPSS version 29 (IBM, Inc). Continuous variables were expressed as mean ± standard deviation and categorical variables as number and percentages of the total group number. Group comparisons were made using the *χ*2 or Fisher's exact test for categorical variables and Student's t-test or one-way analysis of variance for continuous variables. For variables that do not follow a Gaussian distribution, the Mann–Whitney U test was used. A stratified subgroup analysis was performed to assess variables affecting hospital length of stay. We also performed a stratified analysis of grip strength in quartiles. A two-sided *P* value < .05 was considered statistically significant. For multivariate analyses, linear and logistic regression was used to determine if hand grip strength was an independent predictor of continuous and categorical outcomes including variables of univariate significance (*P* < 0.20).

## Results

### Patient characteristics

The baseline characteristics of our study population are listed in Table 1. The mean age was 69 ± 15 years and 37.7% were female. Most common reasons for admission to the CVICU were congestive heart failure (33.3%), acute coronary syndrome (30.9%) and after a scheduled procedure (29.1%). Common comorbidities included congestive heart failure, myocardial infarction, atrial fibrillation, diabetes and chronic kidney disease. Mean albumin level was 2.93 gm/dl and mean lactic acid level was 2.29 mmol/L. Most patients were right hand dominant (85.5%). Peak grip strength was 26 ± 11 kg. Mean CVICU length of stay was 3.8 ± 4.4 days and mean hospital length of stay was 10.5 ± 10.5 days.

**Table 1:** Baseline characteristics.

Characteristic	*N* = 330 (*N*, %)
Mean age (SD) – years	68.6 (14.8)
Body mass index	29.4 (7.4)
Sex (%)	
Female	125 (37.7)
Race (%)	
African American	161 (48.8)
Caucasian	141 (42.7)
Comorbidities (%)	
Congestive heart failure	173 (52.4)
Myocardial infarction	94 (28.5)
Atrial fibrillation	101 (30.6)
Diabetes	112 (34)
Chronic kidney disease	101 (30.6)
Malignancy (active or past)	49 (14.8)
Hyperlipidemia	243 (73.6)
Peripheral vascular disease	37 (11.2)
Chronic lung disease	41 (12.4)
Tobacco use	178 (53.9)
Reason for admission (%)	
Congestive Heart failure	110 (33.3)
Acute coronary syndrome	102 (30.9)
Scheduled procedure	96 (29.1)
Cardiac arrest	16 (14.8)
NYHA functional class (%)	
I	87 (26.6)
II	106 (32.1)
III	97 (29.4)
IV	40 (12.1)
Mean lactic acid (SD) – mmol/L	2.29 (2.31)
Mean albumin level (SD) – gm/dl	2.93 (0.59)
Vasopressor use (%)	84 (25.5)
Inotrope use (%)	77 (23.3)
Mechanical circulatory support	
Intra-aortic balloon pump	41 (12.4)
Impella	3 (0.9)
Mechanical respiratory support	
Noninvasive ventilation	57 (17.3)
Mechanical ventilation	53 (16.1)
Right hand dominance	282 (85.5)
Grip strength (SD) – kg	
Peak	26.23 (11.09)

### Peak hand grip strength and outcomes

**Figure 2:**
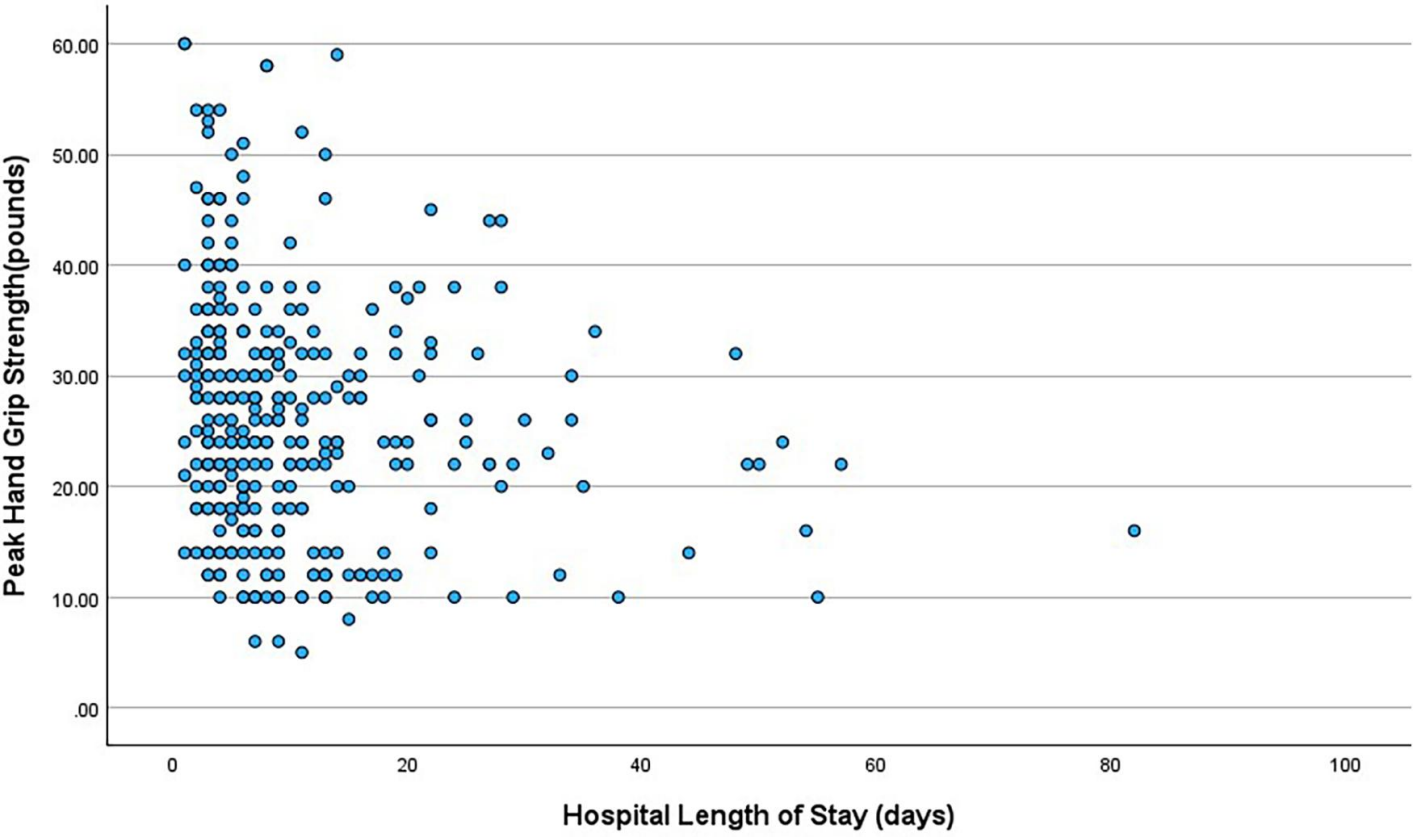
Bivariate correlation between peak hand grip strength and hospital length of stay.

HGS was significantly inversely correlated with hospital LOS (r = −0.165, *P* = 0.003) (Figure 2) but not with CVICU length of stay (r = −0.053, *P* = 0.34). Similar correlation coefficients were observed for subgroups of female and male subjects, although statistically significant only in male subjects due to larger sample size. HGS was only related to hospital LOS in patients with non-procedural CVICU admissions (r = −0.222, *P* < 0.001). There was no relationship among patients with procedural admissions (r = −0.012, *P* = 0.91). Median hospital length of stay was longer among the lowest quartile of HGS (8.5 days; Interquartile range 5–13) compared to the upper quartiles (7 days; Interquartile range 4–12) which was statistically significant (*P* = 0.019) (Table 2). HGS was not associated with either CVICU or 30-day readmission and mortality. Among non-procedural admissions to the CVICU, linear regression identified HGS, age and albumin as strongest predictors of hospital LOS (r = 0.38, *P* < 0.001). Following an elective procedure, the OASIS score (r = 0.426, *P* < 0.001) and albumin (r = −0.380, *P* < 0.001) were better predictors of LOS than HGS (Table 3). In multivariate linear regression, HGS remained a predictor of the primary study endpoint of hospital LOS in non-procedural CVICU admissions.

**Table 2:** Outcomes.

Outcome	Lowest HGS quartile	HGS quartiles 2–4	*P*
CVICU mortality	5 (5.7)	8 (3.3)	0.33
Total in-hospital mortality	7 (8)	13 (5.4)	0.38
CVICU readmission	4 (4.5)	16 (6.6)	0.50
30-day readmission	18 (20.5)	40 (16.6)	0.42
Median CVICU length of stay in days (IQR)	3 (2–5)	2 (1–4)	0.36
Median hospital length of stay in days (IQR)	7 (4–12)	8.5 (5–13)	0.019

CVICU, cardiovascular intensive care unit; HGS, hand grip strength; IQR, interquartile range; SD, standard deviation.

Group comparisons were made using the *χ*2 or Fisher's exact test for categorical variables and Student's t-test or one-way analysis of variance for continuous variables. For variables that do not follow a Gaussian distribution, the Mann–Whitney U test was used.

**Table 3:** Predictors of hospital length of stay for CVICU patients.

Baseline characteristics	Nonprocedural admissions		Patients admitted after scheduled procedure	
	Pearson correlation	*P* value	Pearson correlation	*P* value
Peak grip strength	−0.222	0.01	−0.012	0.91
Age	−0.125	0.56	−0.207	0.04
Body mass index	0.003	0.960	0.000	1.0
Serum albumin	−0.299	<0.001	−0.380	<0.001
Charlson comorbidity points	0.003	0.968	−0.084	0.42
OASIS score	0.205	0.02	−0.426	<0.001

## Discussion

HGS is a validated and safe assessment tool of global muscle function that is included in several frailty scoring systems (11, 14–17) and its feasibility has been demonstrated in multiple studies to date (9, 11, 15–19). HGS correlates well with elbow flexion, knee extension and trunk extension (20, 21) and this allows for assessment of muscle function without placing critically ill, deconditioned patients with poor exertional tolerance at risk of injury from physical exertion (16). Low grip strength is a consistent predictor of death and high grip strength is a consistent predictor of survival in studies with a diverse sample of subjects (22). To our knowledge, this is the first study of assessment of handgrip strength in the CVICU population.

Predicting which patients have risk for long length of stay holds relevance for both patients and to health care organization to maximize resource allocation, develop an effective service plan and improve outcomes. Our study showed an inverse association between HGS and hospital overall hospital LOS. Many other studies have also shown an association of lower hand grip strength with increased likelihood of complications or increased length of stay (22). In a different population of elder medical inpatients, higher admission HGS was associated with increased likelihood of discharge home (23). Potential contributors to the relationship between HGS and LOS include the known meaningful relationship between HGS and both general functional and nutritional status.

In contrast to various population studies that have associated HGS with mortality, we found that hand grip strength was not associated with CVICU or in-hospital mortality. Our findings match those of a study of 167 elderly patients undergoing coronary artery surgery in which frail patients identified using Cardiovascular Health Study frailty index criteria had a longer median length of stay but similar perioperative outcomes (24). It should be noted that in addition to grip strength, Cardiovascular Health study criteria also use weight loss, exhaustion, physical activity and gait speed as markers of frailty. In another study of 110 patients in a surgical ICU setting, manual muscle testing rather than grip strength measurements predicted mortality and length of stay (25) suggesting that global muscle weakness rather than HGS predicts mortality in intensive care unit. Further study of this relationship is warranted to exclude small relationships through larger sample size, and in populations enriched with higher mortality rates.

Because patients admitted emergently to the CVICU with heart failure, arrythmias and cardiac arrest represent a different cohort of population compared to patients admitted for monitoring after an interventional or electrophysiology procedure, we studied predictors of length of stay in both these cohorts. We found that handgrip strength predicted hospital length of stay better in patients admitted for an acute cardiovascular illness compared to those admitted for monitoring in the CVICU after a scheduled procedure. In patients admitted for monitoring after a procedure, OASIS score was a better predictor of hospital length of stay than hand grip strength. Serum albumin, however, was a predictor of length of stay in both groups of patients. A similar relationship of albumin with length of stay was also noted in a study of HGS in a surgical intensive care unit (25). Serum albumin has also been noted to be an independent prognostic tool for predicting mortality and morbidity in surgical patients.

### Study limitations

We found a low event rate experienced by our study sample, consistent with modern CVICU cohorts. It remains possible that in a much larger sample of patients, peak grip strength may be related to mortality as has been seen in many population studies. We used hand grip strength as a single objective measure based upon its simple and accessible nature vs. other markers of frailty. We did not measure hand grip strength before admission and did not conduct longitudinal follow up measurements. Patients requiring significant doses of sedatives and those who have delirium as part of their clinical condition underwent grip strength assessment when deemed suitable by treating physician which may cause heterogeneity in the study. The timing of when to perform grip strength assessments related to sedation or interventions such as mechanical ventilation should be noted as a consideration in clinical translation. We only studied CVICU population of a single tertiary care hospital and this may not be generalizable to other intensive care settings. Finally, use of the highest grip strength value obtained for each patient may have resulted in measurement bias.

## Conclusions

HGS has the potential to be used as a simple, inexpensive, and widely applicable point of care assessment similar to an additional “vital sign” that is understood to objectively quantifies frailty in the critically ill CVICU patient. In this study, we identify the clinical correlates of that relationship, namely that lower values of HGS were independently associated with hospital length of stay among non-procedural patients. Prompt recognition of the frailty phenotype will potentially allow for early and targeted mobilization of often limited resources such as physical and occupational therapy to the most relevant patients in need of interventions that could ultimately reduce ICU length of stay as well as the morbidity and mortality associated with ICUAW.

## Data Availability

The raw data supporting the conclusions of this article will be made available by the authors, without undue reservation.
